# Anti-Cancer Properties of *Stevia rebaudiana*; More than a Sweetener

**DOI:** 10.3390/molecules27041362

**Published:** 2022-02-17

**Authors:** Nikos Iatridis, Anastasia Kougioumtzi, Katerina Vlataki, Styliani Papadaki, Angeliki Magklara

**Affiliations:** 1 Department of Clinical Chemistry, Faculty of Medicine, University of Ioannina, 45110 Ioannina, Greece; iatridisnk@gmail.com (N.I.); natkoug@gmail.com (A.K.); kathy_dv@yahoo.com (K.V.); st.papadaki7@yahoo.gr (S.P.); 2 Biomedical Research Insitute, Foundation for Research and Technology-Hellas, 45110 Ioannina, Greece; 3 Institute of Biosciences, University Research Center of Ioannina (URCI), 45110 Ioannina, Greece

**Keywords:** *Stevia rebaudiana*, antitumor activity, antioxidant, breast cancer, gastrointestinal cancer, cytotoxicity, cancer prevention, bioactive compound

## Abstract

*Stevia rebaudiana Bertoni* is a perennial shrub from Paraguay that is nowadays widely cultivated, since it is increasingly being utilized as a sugar substitute in various foodstuffs due to its sweetness and minimal caloric content. These properties of the plant’s derivatives have spurred research on their biological activities revealing a multitude of benefits to human health, including antidiabetic, anticariogenic, antioxidant, hypotensive, antihypertensive, antimicrobial, anti-inflammatory and antitumor actions. To our knowledge, no recent reviews have surveyed and reported published work solely on the latter. Consequently, our main objective was to present a concise, literature-based review of the biological actions of stevia derivatives in various tumor types, as studied in in vitro and in vivo models of the disease. With global cancer estimates suggesting a 47% increase in cancer cases by 2040 compared to 2020, the data reviewed in this article should provide a better insight into *Stevia rebaudiana* and its products as a means of cancer prevention and therapy within the context of a healthy diet.

## 1. Introduction

Cancer is among the leading causes of death worldwide, accounting for nearly 10 million deaths in 2020 [1]. Global cancer estimates describe an increasing trend in cancer incidence and project a 47% rise in cancer cases by 2040 compared to 2020 [2]. Interestingly, one third of cancer cases are believed to be due to lifestyle risk factors, such as cigarette smoking, heavy alcohol consumption and diet rich in red and processed meat and poor in fruits and vegetables [2]. Thus, health-promoting habits in everyday life, including a plant-based diet, could efficiently lower one’s risk to develop cancer.

**Figure 1:**
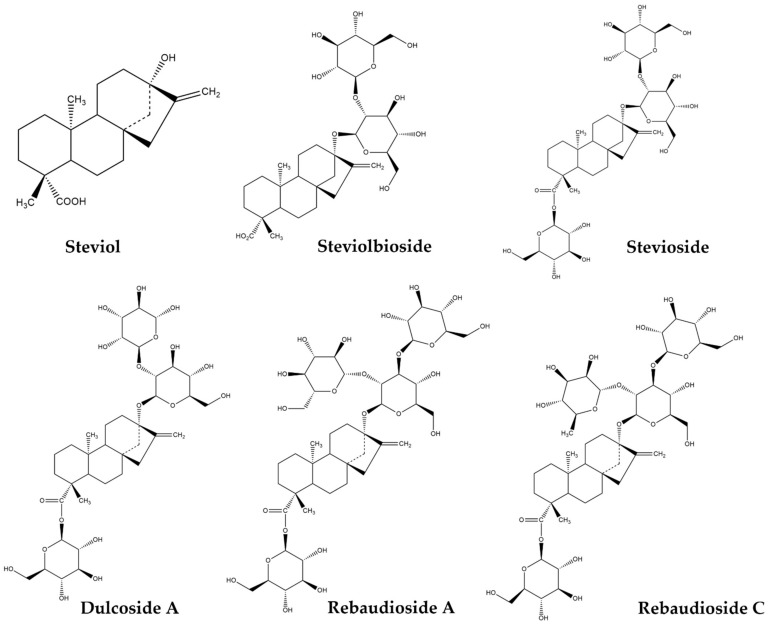
Steviol and SGs from the plant *Stevia rebaudiana Bertoni*. Steviol is the core aglycone of the glycosides. Stevioside, Rebaudioside A and Rebaudioside C are the most abundant glycosides. Dulcoside A is described in studies reviewed in this paper. Steviolbioside is a hydrolysis product of Stevioside often used in anti-cancer studies. (Structures were designed using Chem Draw Ultra).

*Stevia rebaudiana Bertoni* (or simply called Stevia), a perennial shrub from South America and indigenous to Paraguay, is widely known as a natural sweetener, which is 300–450 times sweeter than sucrose. Several reports confirm that Stevia also exhibits several biological effects valuable to human health [3,4,5]. Stevia consumption appears to have positive outcomes in chronic diseases, such as hyperglycemia, dyslipidemia and hypertension, while numerous studies describe its antioxidant, anti-inflammatory and anti-cancer effects [3,4,5]. These properties are attributed to the plant’s leaf extracts [6,7,8,9], which contain secondary metabolites, such as steviol glycosides (SGs), [10,11] and polyphenols [12] with potentially important bioactive effects. SGs are responsible for the plant’s sweet taste and are classified as ent-kaurane type diterpenes with a distinct chemical structure composed of sugar moieties attached to an aglycone named Steviol (Figure 1). More than 40 SGs have been identified so far, with the most abundant being Stevioside, Rebaudioside A and Rebaudioside C (Figure 1) [13].

Apart from contributing to the plant’s sweetness, research has shown that the SGs as well as various stevia extracts demonstrate potent antitumor activities. In this review, we have surveyed the literature to compile the results from such studies and provide a succinct summary that will be helpful in integrating current knowledge on this topic, but also in pinpointing weaknesses in the study design and ways to overcome them. Given that breast and gastrointestinal (GI) carcinomas were among the top five most diagnosed as well as most deadly malignancies in 2020 [1], we have mainly focused on stevia-related studies in these tumor types.

## 2. Steviol Glycosides: Chemical Structure and Metabolism in the Human Body

**Figure 2:**
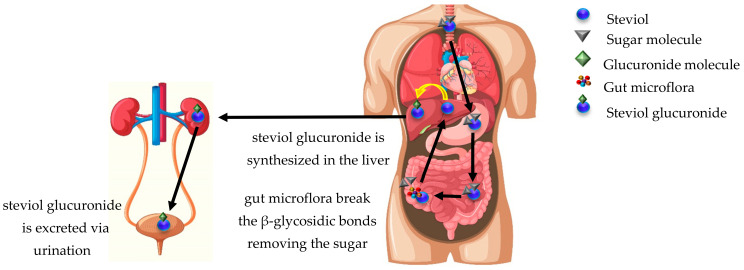
SG metabolism in the human body. Steviol with sugar molecules attached to it enters the body, but it cannot be metabolized by the components of the upper GI track. SG metabolism starts in the large intestine, where gut microflora breaks the β-glycosidic bonds removing the sugar molecules, leaving the core steviol to be transported to the liver via the hepatic portal vein. In the liver, a glucuronide molecule is attached to steviol, leading to the formation of steviol glucuronide, which is subsequently transported to the kidneys via systemic circulation and is finally eliminated via urination. (The outline of the human body was reproduced from www.freepik.com, accessed on 20 December 2021).

As mentioned above, SGs are classified as ent-kaurene-type diterpenes with sugar moieties attached to the aglycone steviol. These sugar moieties can be either glucose, rhamnose, xylose, fructose or deoxyglucose, constituting five distinct SG families [13]. The presence of β-glycosidic linkages between steviol and the sugar groups is an important factor for the SG’s metabolism. It is now established that once an SG is administered orally, it cannot be metabolized by enzymes, such as salivary and pancreatic α-amylase, pepsin and pancreatin found in saliva and gastric secretions of the upper GI tract [11,14,15,16]. As shown in Figure 2, SGs metabolism and breakage of their β-glycosidic bonds is carried out in the large intestine by the gut microflora [3,17], a process that lasts 10 to 24 h depending on the SG being digested [18]. As the sugar molecules are removed, the common chemical core steviol that remains is resistant to bacterial digestion in the colon [19] and is transported to the liver via the hepatic portal vain, where a glucuronide molecule gets attached to it. The newly synthesized steviol glucuronide is transported via the systemic circulation to the kidneys and is, ultimately, excreted from the body via urination. Remarkably, the sugar moieties do not enter the bloodstream and are used by the gut microflora as an energy source [3,17]. This property of SGs has led to their classification as non-caloric sweeteners by food administration authorities across the world [15,20].

## 3. Antitumor Effects of *Stevia rebaudiana* Derivatives

SGs metabolism renders stevia consumption a potentially healthy choice that may contribute to reduced glucose levels in the blood circulation resulting in a number of health benefits, as mentioned before [3,4,5,6,7,8] and depriving tumor cells from an essential source of energy. In parallel, the plant derivatives manifest cytotoxic and anti-proliferative effects in cancer cells and in vivo tumor models, as recent research suggests. An overview of the most important data from published literature will be discussed in the following sections.

### 3.1. Antitumor Effects of Stevia rebaudiana Derivatives in Breast Cancer

Breast cancer has risen to be the most commonly occurring malignancy worldwide with growing evidence indicating that lifestyle factors, including diet, may be associated with a higher risk [1,2]. A nutritional plant-based intervention, as part of a preventive or therapeutic approach against the disease, may contribute to reducing cancer incidence and improving patient outcome [2]. Based on the studies discussed in this section, stevia products may be valuable components of such a diet.

Early studies regarding *S. rebaudiana* metabolites were conducted in vivo using rodent models and investigating the adverse effects of these compounds. One such study was performed by Toyoda et al. in F344 rats, who examined the potential carcinogenic effects of stevioside, the most abundant steviol glycoside in the stevia leaf [21]. The rats underwent a 108-week modified diet with two different stevioside concentrations (2.5% and 5%). At the end of the study, the researchers found no significant differences in tumor incidence between the stevioside-treated and control groups, confirming the glycoside’s lack of carcinogenicity. Interestingly, the frequency of spontaneous mammary adenomas in female rats was actually decreased in the stevioside-treated groups [21], underlining its potential role as a cancer preventive agent. 

Subsequent studies, reporting on the anti-proliferative and cytotoxic effects of stevia derivatives in various breast cancer cell lines, confirmed these initial results. 

The work by Gupta et al. showed that steviol, the core aglycone of SGs, exhibited a potent cytotoxic effect in the MCF-7 breast cancer cells [22]. Steviol induced a dose-dependent decline in cell viability, with the cell population reduced to 40%, when treated at the highest concentration (500 μM) for 48 h. Cell cycle analysis showed that steviol caused an arrest at the G2/Μ phase transition, while it also induced a reactive oxygen species (ROS)-mediated apoptosis [22]. 

Similar cytotoxic results in this cell line were obtained by treatment with stevioside, which exhibited a time- and dose-dependent effect, as reported by a different group [23]. The anti-proliferative effects of stevioside, however, were achieved within a lower range of concentrations (2.5–30 μM) compared to steviol [22], arguing that the glycoside is a more potent anti-cancer compound. The cell cycle studies conducted to explore the mechanisms driving stevioside’s cytotoxicity showed a cell population increase in the G0/G1 phase. The researchers noted that the cells were neither in a replication nor in a resting phase, hypothesizing that cellular death could be taking place. Annexin V staining confirmed that cells were undergoing a ROS-mediated apoptosis, when treated with a relatively low concentration of stevioside (10 μM). This effect was more pronounced 72 h post-treatment with apoptotic cells reaching ~70% of the total population [23]. 

**Figure 3:**
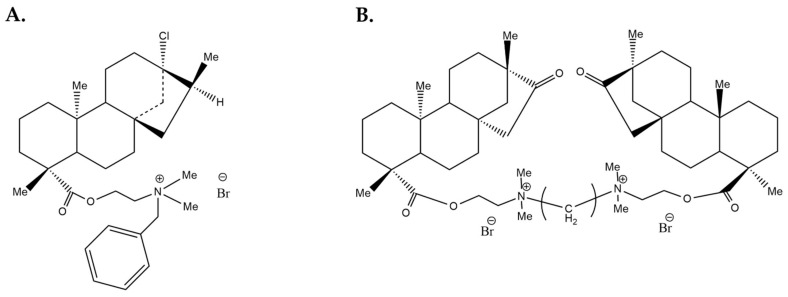
Chemical structures of (**A**) mono-quaternized derivative of steviol and (**B**) bis-quaternized derivative of isosteviol. Reprinted from [24] with permission from Elsevier (Structures were designed by using ChemDraw Ultra (PerkinElmer, Waltham, MA, USA)).

Ammonium derivatives of steviol and isosteviol were also investigated in MCF-7 cells regarding their cytotoxic effects [24]. Isosteviol is a hydrolysis product of stevioside. The results of this study were of particular interest, as the authors showed that two of the synthesized compounds, a mono-quaternized derivative of steviol and a bis-quaternized derivative of isosteviol (shown in Figure 3) exhibited a cytotoxic effect (IC_50_ 5 μM) in the MCF-7 cells, which was as potent as that of the anti-cancer drug doxorubicin (IC_50_ 3 μM). Even more remarkably, the two compounds had a selective effect on cancer cells, as they were less toxic in the Wi38 human embryonic lung cell line, as opposed to the drug. Annexin V staining of treated MCF-7 cells confirmed that the cytotoxicity of the two compounds was mainly due to induction of apoptosis [24]. Further experiments studying the effects of the compounds on the mitochondrial membrane potential and the expression of early apoptosis markers suggested that the induced apoptotic process proceeded along the internal (mitochondrial) pathway [24].

Similar anti-cancer effects of stevia metabolites were also described in the cell lines of other breast cancer molecular subtypes. 

In a study conducted by Khare et al., the triple-negative MDA-MB-231 and the HER2^+^ SKBR-3 breast cancer cells were treated with stevioside with concentrations ranging between 5–100 μΜ for 48 h [25]. This led to a reduction in cell viability by ~60% in both cell lines. Moreover, the glycoside was able to enhance the chemosensitivity of these cells to the common anti-cancer drug 5-Fluorouracil (5-FU). Investigation of the underlying mechanism revealed that combined administration of the two compounds induced an increase in the Bax/Bcl-2 protein ratio and in the apoptotic process via DNA fragmentation, as compared to monotreatment with 5-FU or stevioside [25]. The Bcl-2 protein family members play an important role in promoting or inhibiting intrinsic apoptotic pathways triggered by mitochondrial dysfunction and therefore, the balance between them determines cellular fate [26]. Bax is responsible for cell death promotion, whereas Bcl-2 prevents apoptosis by inhibiting the activity of Bax. When the Bax/Bcl-2 protein ratio is increased, it means that Bax is overexpressed triggering cellular death via apoptosis [26].

The MDA-MB-231 breast cancer cells were also found to be sensitive to other Stevia metabolites, such as steviolbioside [27] and isosteviol derivatives [28]. Chen et al. [27] introduced a novel way of synthesizing steviolbioside (shown in Figure 1), a rare sweetener found in the stevia leaf, by utilizing β-galactosidase in a packed bed bioreactor for an optimal production yield. This enzyme was shown to specifically hydrolyse stevioside into steviolbioside. A packed bed bioreactor consists of one or more tubes packed with catalyst particles of various morphologies and operated in a vertical position [29]. Enzymes can be immobilized on the catalyst particles and fluid can flow through them [29]. In this case, β-galactosidase was immobilized onto crosslinked chitosan microspheres and a stevioside aqueous solution flowed in the reactor coming in direct contact with the catalyst particles, where the glycoside’s hydrolyzation occurred, producing steviolbioside [27]. The anti-proliferative effects of steviolbioside and stevioside were subsequently evaluated in cell lines of different tumor types including the MDA-MB-231 one, while the anti-cancer drug 5-FU was used as a positive control. After a 48-h treatment at a concentration of 250 μg/mL, 5-FU (1.92 mM), steviolbioside (0.39 mM) and stevioside (0.31 mM) led to a 70%, 35% and 20% cell growth inhibition, respectively [27]. Hence, steviolbioside was deemed to be a more effective antitumor compound than its starting material. However, the authors also showed that steviolbioside was more toxic to normal cells than stevioside raising concerns about its safe use [27].

**Figure 4:**
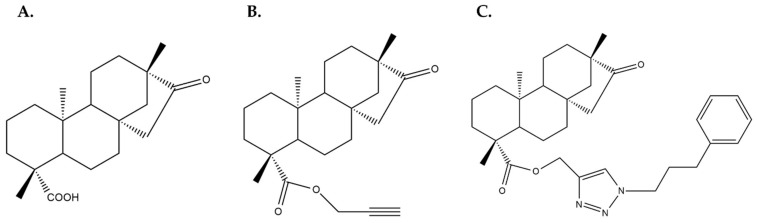
Chemical structures of (**A**) Isosteviol, (**B**) Isosteviol with a modified carboxyl group and (**C**) an Isosteviol triazole conjugate attached to a benzene ring, as described in [28]. (Structures were reproduced from the original study using ChemDraw Ultra).

In a different study, the authors designed and synthesized a wide variety of isosteviol derivatives conjugated with a triazole group [28]. Alkyne isosteviol derivatives were synthesized by modifying isosteviol’s carboxylic (Figure 4B) or ketone group and these compounds were used to construct isosteviol triazole. The latter contained a 1,2,3-triazole group attached to an aromatic ring conjugated to isosteviol’s modified carboxylic group. The most potent of these compounds (depicted in Figure 4C) exhibited an IC_50_ value of 13.76 μM in the MDA-MB-231 breast cancer cells [28]. It should be mentioned that the anti-cancer activity of various 1,2,3-triazole hybrids and conjugates in different human cancer cell lines has been confirmed by other studies as well, highlighting them as novel therapeutic candidates [30].

Besides the cytotoxic effect of purified glycosides, it has been reported that stevia leaf extracts exhibit similar anti-cancer actions, albeit less potent. Stevia extracts vary depending on the organic solvent and the method used for their production. These parameters can alter the composition of the resulting extract and impact its biological activity. A biologically important fraction of the extract that is affected is the total polyphenol content [31] that largely determines its cytotoxicity and antioxidant capacity, as will be discussed later. 

To determine the impact of distinct solvents on the cytotoxic potential of stevia extracts, Ibrahem et al. used acetone, chloroform, water, ethanol, petroleum ether and methanol to generate six different extracts and they tested them in MCF-7 cells [32]. The extracts were produced by directly mixing dried stevia leaves with each solvent and underwent a filtration and dryness process before being used in subsequent experiments. All six extracts succeeded in decreasing cell growth with IC_50_ values ranging from 79 to 374 µg/mL (Table 1) with the petroleum ether extract being the most effective one [32]. 

Interestingly, as Table 1 indicates, there was a reverse correlation between the solvent’s polarity and the extract’s potency. This finding suggests that the most effective bioactive metabolites in stevia are non-polar and better extracted using a solvent of the same nature, such as the petroleum ether. The latter is a commonly used laboratory solvent that is suited for the extraction of hydrocarbons and lipids. In this study [32] the authors did not provide an analysis of the extracts’ constituents; however, other groups have used this solvent to extract ent-kaurane diterpenoids from various plants [34,35]. Consequently, we can assume that the petroleum ether extract from the stevia leaves used in this study was rich in SGs that were responsible for the anti-proliferative effects observed.

An hydro-alcoholic stevia extract produced by a different group via a maceration protocol was also tested in MCF-7 breast cancer cells [36]. The cells were treated for 72 h with extract concentrations ranging between 1–1000 μg/mL and cell viability was reduced by 50% at the highest concentration used. Furthermore, the IC_50_ value of the hydro-alcoholic extract was compared to that of the commonly used anti-cancer drug cisplatin (used at 0.53 mM). Despite having a significantly higher IC_50_ value (98.82 μg/mL vs. 15.96 μg/mL), as expected, the stevia extract was deemed to be a potent complementary therapeutic modality against cancer [36].

In an interesting study by Vaško et al., the antioxidant capacity of four plant extracts (oregano, sage, ginseng and stevia) was linked to their cytotoxic effects in cancer cell lines [37]. A dried root ethanolic extract from ginseng, an aqueous extract from stevia leaves and essential oils extracted from oregano and sage were used. Cell viability was mostly impacted by the oregano and sage extracts due to their ability to affect the mitochondrial redox state and establish a nitrosensitive stress responsible for apoptosis [37]. In contrast, the stevia extract did not have such an effect, although it did exhibit antioxidant properties. Cell viability assays in MCF-7 and MDA breast cancer cells showed that the stevia extract had little to no cytotoxic effect, despite the fact that it reduced the cell populations down to 15.57% and to 13.59%, respectively, 72 h post-treatment with the highest concentration (1000 μg/mL) [37]. As the authors reported no cell death, it is safe to assume that the reduced cell viability was probably due to a potent cytostatic effect of the extract.

The use of nanotechnology in drug delivery for cancer therapy has exploded in recent years [38]. Nanoparticles (NPs) have shown great potential in reducing drug toxicity and side effects; however, it has been recently recognized that carrier systems themselves may inflict damage to the patients. Thus, natural compounds that are expected to be less harmful are increasingly utilized in the manufacture of NPs. Green synthesis is based on the use of plant-derived, environmentally friendly, non-hazardous materials for NP synthesis [39]. For example, plant extracts can serve as capping agents used to coat NPs [40,41,42]. These stabilizing agents constitute a critical parameter in NP synthesis, as they can alter their biological effects and physio-chemical characteristics [40]. Moreover, capping agents are nowadays considered as novel therapeutic agents in combination with the biocompatible NPs they have been attached to. The use of plant-based capping agents is considered to reduce intracellular toxicity, while enhancing biocompatibility and bioavailability of NPs [40,41]. In this context, an aqueous extract generated from dried stevia leaf powder was used to coat ZnS NPs with steviol glycosides [42]. The NPs were administered to MCF-7 cells and their IC_50_ value was determined to be 400 μg/mL, when the aqueous extract alone had a 5-fold higher IC_50_ value, leading the authors to conclude that such NPs could be used as an effective therapeutic means for anti-cancer drug delivery [42].

In conclusion, *S. rebaudiana* derivatives including the core aglycone steviol, SGs and various extracts, exhibit anti-proliferative effects in breast cancer cells of luminal A, HER-2^+^ and triple-negative tumor subtypes, although a very limited number of such cell lines have been examined. In the studies reviewed above and also presented in Table 2, the most commonly used stevia extract was the aqueous one; however, its IC_50_ values in MCF-7 cells varied significantly [32,37,42], highlighting the difficulty in comparing the biological activity of extracts produced by different groups, even when the same solvent is used. Further studies using standardized protocols regarding extraction methods and cell viability assays could elucidate the exact anti-cancer capabilities of each type of extract. Detailed characterization of the extracts’ composition by chromatographic and/or spectroscopic techniques is also an important parameter, which was missing from most studies.

### 3.2. Antitumor Effects of Stevia rebaudiana Derivatives in Gastrointestinal Cancer Models

The number of GI cancer cases, and especially those of colorectal cancer, has significantly risen in developed countries in recent years. Poor diet and obesity have been implicated as high-risk factors and a plethora of reports have emphasized the role of plant-based nutrition in providing protection against these tumor types. Along these lines, several studies have examined the biological effects of *S. rebaudiana* derivatives in GI cancer models including gastric, colorectal and pancreatic cancer, as well as hepatocellular carcinoma and are discussed below and summarized in Table 3. 

Steviol was demonstrated to have anti-proliferative effects in six different human GI cancer cell lines including the gastric MKN-45, MGC-803 and HCG-27 and the colorectal HCT116, HCT-8 and Caco-2 cells [43]. Cytotoxicity assays indicated that steviol inhibited cell viability across all cell lines in a dose-dependent manner, with the gastric cancer cells being more sensitive to treatment compared to the colorectal ones. Remarkably, when cells were treated with 100–200 μg/mL (0.31–0.63 mM) of steviol, its effects on cell growth (60–90% inhibition) were comparable to those of 100–200 μg/mL (0.77–1.54 mM) of 5-FU. To investigate the mechanisms of steviol-mediated cytotoxicity, the authors performed cell cycle analysis and demonstrated that steviol caused a G1 phase arrest in the MKN-45, HGC-27, HCT-116 and Caco-2 cells and a G2 phase arrest in the MGC-803 and HCT-8 cells [43]. 

**Table 3:** Biological effects of *S. rebaudiana* derivatives in gastrointestinal cancer models.

Cell Line/Cancer Type	Compound	Effect (Cell Viability Assay)	Reference
AZ521 (Gastric)	Steviol, isosteviol derivatives	Increased cytotoxicity (MTT Assay)	[44]
HGC-27 (Gastric)	Steviol	Increased cytotoxicity, G1 arrest (MTT Assay)	[43]
MKN-45 (Gastric)	Steviol	Increased cytotoxicity, G1 arrest, apoptosis, regulation of miR-1268b and miR-23c (MTT Assay)	[43]
MGC-803 (Gastric)	Steviol	Increased cytotoxicity, G2 arrest (MTT Assay)	[43]
NUGC-3 (Gastric)	Isosteviol	Increased cytotoxicity (MTT Assay)	[45]
Caco-2 (Colorectal)	Aqueous extract	Decreased cell viability (MTT Assay)	[37]
	Steviol	Increased cytotoxicity, G1 arrest (MTT Assay)	[43]
HCT116 (Colorectal)	Isosteviol Triazole Conjugates	Increased cytotoxicity (MTT Assay)	[28]
	Stevioside, ethanolic extract	Increased cytotoxicity (MTT Assay)	[46]
	Steviol	Cell proliferation inhibition, G1 arrest, apoptosis regulation of miR-203a-3p and miR-6088 (MTT Assay)	[43]
HCT-8 (Colorectal)	Steviol	Cell proliferation inhibition, G2 arrest (MTT Assay)	[43]
Hep3B (Hepatocarcinoma)	Steviolbioside	Cell proliferation inhibition (MTT Assay)	[27]
HepG2 (Hepatocarcinoma)	Aqueous extract, stevioside, RebA	No cytotoxicity (LDH and BRDU assays)	[10]
	Commercialized stevia, Stevioside	Increased cytotoxicity, cholesterol internalization (MTT Assay)	[47]
	Ammonium derivatives of steviol and isosteviol	Increased cytotoxicity (Multifunctional Cytell Cell Imaging system)	[24]
ASPC-1 (Pancreatic)	Isosteviol Triazole Conjugates	Increased cytotoxicity (MTT Assay)	[28]
BxPC-3 (Pancreatic)	Steviolbioside	Cell proliferation inhibition (MTT Assay)	[27]
MiaPaCa-2 (Pancreatic)	Stevioside, ethanolic extract	Increased cytotoxicity (MTT Assay)	[46]

Western Blot assays confirmed that p21 and p53 were upregulated and cyclin D was downregulated in the former cells establishing the G1 arrest. The authors also showed that an apoptotic process was induced in the ΜΚΝ-45 and HCT-116 cells upon steviol treatment by utilizing Annexin V-FITC/PI double-labeled flow cytometry. Western Blot analysis verified an increase in the Bax/Bcl-2 apoptotic protein ratio, indicating a mitochondria-mediated apoptosis. Finally, the researchers investigated the expression of several miRNAs, implicated in the deregulation of crucial cell processes in cancer, such as cell proliferation, differentiation and apoptosis. Their analysis showed that steviol treatment altered the expression of miR-203a-3p and miR-6088 in HCT-116 and that of miR-1268b and miR-23c in MKN-45 cells, presumably, inducing growth inhibition [43].

Mizushina et al. studied the cytotoxic effects of steviol, stevioside, isosteviol and isosteviol derivatives on various cancer cell lines, including the gastric cells NUGC-3 [45]. Isosteviol inhibited cell growth after a 48-h treatment reducing cell viability by 60%, while the other compounds did not exhibit cytotoxic effects [45]. To gain a mechanistic view of this effect, the authors studied the functional properties of isosteviol regarding the inhibition of DNA polymerase and topoisomerase activities. Isosteviol was capable of inhibiting the mammalian DNA polymerase λ and also the human DNA topoisomerase II in vitro with IC_50_ values of 103 μM and 177 μM, respectively. Furthermore, fluorescence studies with ethidium bromide (EtBr), a compound that intercalates with dsDNA, and isosteviol showed no changes in the EtBr emission spectra, indicating that isosteviol does not bind to DNA. Kinetic studies of the above-mentioned enzymes suggested a decrease in enzymatic activity with the presence of isosteviol. Of note, human topoisomerase II activity was also hindered by stevioside and 17-hydroxyisosteviol with IC_50_ values of 90 μM and 82.5 μM, respectively [45]. These findings altogether suggested a possible mechanism for the cytotoxic action of these stevia metabolites in cancer via inhibition of enzymes crucial for DNA replication.

**Figure 5:**
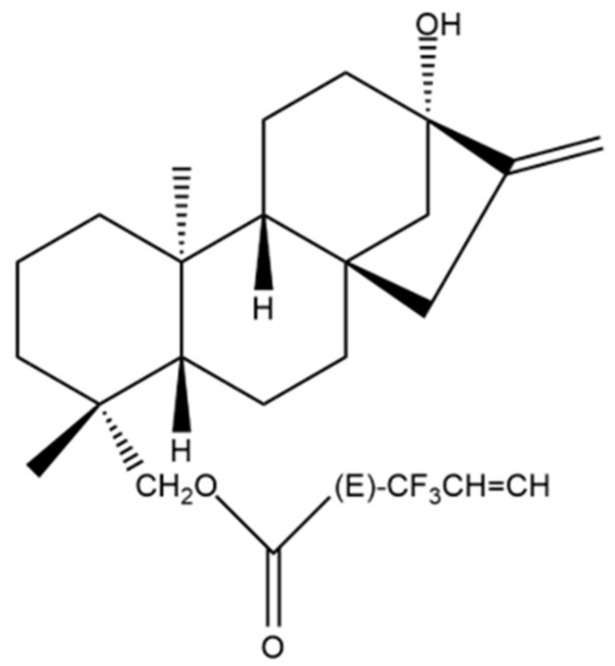
Chemical structure of the steviol 19-O-acylated derivative ent- 454 kaur-16-ene-13,19-diol 19-*O*-4′,4′,4′-trifluorocrotonate. Reprinted from [44] with permission from Wiley. (Structure was designed by using ChemDraw Ultra).

In a later study performed by Ukiya et al., the biological effects of 17 steviol and 19 isosteviol derivatives were also examined in various cancer cell lines including the stomach cancer cell line AZ521 [44]. In this study, the compounds generated from isosteviol and steviol underwent various modification reactions to produce acylated, ester, alcoholic and carboxyl acid derivatives. Out of the 36 compounds tested, the steviol 19-*O*-acylated derivative ent-kaur-16-ene-13,19-diol 19-*O*-4′,4′,4′-trifluorocrotonate (depicted in Figure 5) was the most cytotoxic in all cell lines, with an ΙC_50_ value of 1.7 μM in the AZ521 cells [44].

Other isosteviol derivatives tested for their anti-cancer activity in GI cancers include the ones conjugated with a triazole group designed and synthesized by Khaybullin et al. (also discussed in the previous section) [28]. The same compound that exhibited a potent cytotoxic effect against the breast cancer MDA-MB-231 cells (depicted in Figure 4C) was also cytotoxic in the colon cancer cell line HCT166 and the pancreatic cancer cell line ASPC-1 with remarkably low IC_50_ values (4.79 μM and 6.60 μM, respectively) [28]. 

The effect of steviolbioside was studied in a number of cell lines of different tumor types and the authors demonstrated a notable growth inhibition in the hepatocellular carcinoma Hep3B and the pancreatic cancer BxPC-3 cell lines, similar to that observed in the MDA-MB-231 cells described in the previous section [27]. This metabolite exhibited a >3-fold higher anti-cancer activity than stevioside in both GI cell lines, when the two compounds were administered at 0.39 mM and 0.31 mM (250 μg/mL), respectively, for 48 h [27]. However, as it was noted before, the fact that steviolbioside was more toxic to normal cells than stevioside poses questions about patient safety.

Several studies have also investigated the biological effects of *S. rebaudiana* extracts in GI cancer cell lines, as discussed next.

The four different plant extracts (oregano, sage, ginseng and stevia) used by Vaško et al. and discussed earlier (see Section 3.2) were also evaluated in the human colorectal cancer cell line Caco-2 [37]. Similarly to what was observed in the MCF-7 breast cancer cells, the stevia aqueous extract did not demonstrate cytotoxic effects, but it notably reduced the viability of the colon cells down to 6%, when used at the highest concentration (1000 μg/mL) [37].

Lopez et al. examined the antioxidant capacity and cytotoxic effects of a *S. rebaudiana* ethanolic extract in comparison to stevioside in the GI human cancer cell lines HCT116 (colorectal) and MiaPaCa-2 (pancreatic) [46]. The antioxidant activity of the ethanolic extract was measured by the DPPH (2,2-diphenyl-1-picryl-hydrazyl-hydrate) assay and the xanthine-xanthine oxidase system. Both assays showed a potent free radical scavenging capability for the extract but not for the glycoside. The lack of antioxidant activity was attributed to the absence of phenyl aromatic rings in stevioside’s structure, which are responsible for the free radical scavenging capability. As the authors noted, the extract contained a respectable amount of polyphenols (2.2%), which could exert the antioxidant activity observed [46]. In terms of cytotoxicity, both the ethanolic extract and the purified stevioside showed a dose-dependent anti-proliferative effect in the cancer cells lines [46]. The phytochemical analysis of the extract showed that it contained 28% of SGs. Since the viability assays produced similar results, the authors concluded that the stevia extract was more potent than the glycoside regarding cytotoxicity, probably due to the presence of other substances with anti-proliferative properties; this was also supported by further experiments. The authors studied a key enzyme of cell cycle regulation, Cyclin Dependent Kinase–4 (CDK4) and found that it was inhibited by the stevia extract but not by stevioside [46]. Since the cyclin D1–CDK4 complex is crucial for cell cycle S phase entry (G1 checkpoint), this finding could explain the anti-proliferative effects of the extract. CDK4 inhibition was attributed to the presence of polyphenols in the extract, a hypothesis supported by various studies on polyphenolic compounds [48,49,50,51,52].

The antioxidant and cytotoxic properties of various *S. rebaudiana* extracts were also evaluated in the hepatocellular carcinoma HepG2 cells [10]. All extracts were generated from commercially available *S. rebaudiana* dry stevia leaves or stems powder. The samples used included dry leaves powder, dry leaves powder from organic farming, two different types of dry leaves, dry leaves from Peru and dry stem powder from Peru. The extracts were prepared by infusing stevia powder in distilled water at 100 °C at a concentration of 10 g/L. The different extract solutions were analyzed in terms of total polyphenol and steviol glycosides content. The dry leaves extracts (except the ones from Peru) had an SG content ranging from 10.9% to 14.3%, while the polyphenol content was more variable [10]. Notably, the dry leaves powder from organic farming had a higher polyphenol content compared to the non-organic one. The purified SGs, stevioside and rebaudioside A, were also included in the study. The antioxidant capacity of the extracts was evaluated using the oxygen radical absorbance capacity assay, which measures the radical chain breaking ability of antioxidants by monitoring the inhibition of peroxyl radical-induced oxidation [53]. Peroxyl radicals are the main free radicals found in lipid oxidation in foods and biological systems under physiological conditions [53]. The results of this assay showed a good correlation with the results obtained by a cellular antioxidant activity assay, assessing the extracts’ intracellular antioxidant capacity in HepG2 cells. In conclusion, the extracts demonstrated a remarkable antioxidant capacity in scavenging peroxyl radicals at the intracellular level [10]. Comparing leaf and stem extracts, the latter presented a lower antioxidant capacity, while no antioxidant activity was exhibited by the purified steviol glycosides. The cytotoxicity of the stevia extracts in HepG2 cancer cells was evaluated by the lactate dehydrogenase (LDH) assay after treating cells with 1 mg/mL of each extract. The LDH assay is based on the release of lactate dehydrogenase into the culture medium as a result of cellular membrane damage, upon treatment with toxic concentrations of a sample substance. Measuring the absorbance at 490 nm allows the quantification of cellular toxicity. All the extracts showed minimal growth inhibition, ranging between 2.2–9.9%, while the purified glycosides exhibited no cytotoxic effects, even at higher concentrations [10]. The cytotoxicity of the extracts was also examined by the BrdU colorimetric assay, confirming the absence of any effects on cell proliferation [10]. It is noteworthy that the antioxidant properties of the stevia extracts, as verified in this study, are in accordance with the results of other published work discussed in this section [37,46]. These findings highlight the potent antioxidant capability of *S. rebaudiana* extracts, which could be a key factor in managing oxidative stress-related conditions, ultimately protecting cells from undergoing molecular changes that could trigger carcinogenesis [54].

The HepG2 hepatocarcinoma cell line was also used to evaluate the lipid regulation efficacy and cytotoxicity of a commercialized stevia extract powder and stevioside [47]. The cancer cells were treated with these compounds for 24 h with a wide range of concentrations (extract 0.5–20 mg/mL, stevioside 1–10 μM), which are notably higher for the extract compared to other studies [32,36,37,46]. The MTT assay showed a dose-dependent cytotoxicity for both compounds and the IC_50_ was calculated at 8.68 mg/mL for the extract and at 10.91 µM for the glycoside [47]. Regarding lipid regulation, both compounds were able to trigger internalization of cholesterol in HepG2 cells [47]. This observation was confirmed by immunofluorescence imaging of intracellular lipid droplets and of the low-density lipoprotein receptor, suggesting that cholesterol was indeed stored intracellularly. It was also proposed that stevia derivatives interfered with the mevalonate pathway, preventing HMG-CoA conversion to mevalonate, a crucial step in cholesterol synthesis [47]. Thus, treatment with stevia derivatives would reduce cholesterol level, which could be linked to their anti-cancer activity, since increased cholesterol synthesis is a hallmark of cancer, responsible for membrane biogenesis, cancer stemness preservation and tumor cell energy needs [55]. Impairing cholesterol metabolism via the mevalonate pathway has been characterized as a potent antitumor strategy [55] and stevia products could be capable of accomplishing this.

In conclusion, the above studies confirm the anti-proliferative effects of SGs in GI cancers, but also highlight the potency of stevia’s extracts through their antioxidant and lipid regulation capabilities. Further studies could possibly introduce novel, next-generation, cancer prevention and therapeutics modalities based on the above findings.

### 3.3. Antitumor Effects of Stevia rebaudiana Derivatives in Other Solid Tumors and Blood Cancers

An early study investigated the effects of the purified SGs stevioside, rebaudiosides A and C and dulcoside A in mouse skin tumor formation [56]. The glycosides inhibited, in a dose-dependent manner, the inflammatory and tumorigenic activities of 12-O-tetradecanoylphorbol-13-acetate (TPA), a chemical compound that promotes tumorigenesis and is commonly used as an artificial inducer of inflammation. A similar in vivo study examined the effects of steviol, stevioside and isosteviol in papilloma formation in mice using two-stage carcinogenesis assays [57]. The authors used either 7,12-dimethylbenz[a]anthracene (DMBA) or peroxynitrite for tumor initiation and TPA for tumor promotion [57]. All three SGs exhibited a strong tumor inhibitory effect in both assays showing promise as cancer preventive agents [57]. Of note, it was also shown that the stevia metabolites were more potent than glycyrrhizin, which is known for its antitumor-promoting activity in chemical carcinogenesis [58].

The anti-cancer activity of steviol was also assessed in the U2OS osteosarcoma cell line [59]. Steviol exhibited a dose- and time-dependent inhibitory action on the proliferation of these cells, as it was demonstrated by a decrease in their colony formation ability. Strikingly, the potency of steviol’s effect was comparable to that of 5-FU with their IC_50_ values being 200 μg/mL (0.63 mM) and 250 μg/mL (1.92 mΜ), respectively, albeit much weaker than doxorubicin, which had an IC_50_ of 1.2 μg/mL (2.2 μΜ) [59]. Τhe authors also investigated the mechanisms driving the steviol-mediated inhibition and showed that the compound promoted G1 cell cycle arrest by up-regulating the G1-associated proteins p21, p53, cyclin E and CKD2 and downregulating cyclin D. Additionally, they found that the apoptosis-associated protein Bax-1 was increased, whereas Bcl-2, caspase 3 and survivin were decreased, leading them to conclude that steviol induced a mitochondrial apoptotic pathway independent of survivin and caspase 3 [59]. 

A number of studies discussed in the previous sections regarding the cytotoxic effects of SG derivatives in breast and/or GI cancer cell lines also included other tumor types and will be briefly presented. The panel of isosteviol triazole conjugates developed by Khaybullin et al. (see Section 3.1 and Section 3.2) were also evaluated for their anti-cancer effects in lung (A549), cervical (HeLa) and leukemic cells (MOLT-4 and HL-60), where they showed significant inhibition of cell growth [28]. In the study by Ukiya et al. [44] (also see Section 3.2), the cytotoxic and antiapoptotic effects of the 36 steviol and isosteviol derivatives were also evaluated in leukemia (HL60) and lung (A549) cancer cell lines, confirming that the most effective cytotoxic compound was the steviol 19-*O*-acylated derivative ent-kaur-16-ene-13,19-diol 19-*O*-4′,4′,4′-trifluorocrotonate (Figure 5). This compound was also gauged in the leukemic HL60 cells, where it was shown that it induced typical apoptotic death, as revealed by flow cytometric analysis using an antibody against annexin V and propidium iodide [44]. The isosteviol derivatives developed by Mizushina et al. and tested in NUGC-3 gastric cells (see Section 3.2) were also examined in two human leukemia cell lines (MOLT-4 and BALL-1) yielding similar results, i.e., only isosteviol resulted in growth inhibition in a dose-dependent manner [45]. The ammonium derivatives of steviol and isosteviol tested in MCF-7 and HepG2 cells (see Section 3.1 and Section 3.2, respectively) were also used in cervical (M−HeLa), lung (A549) and prostate (PC3) cancer cell lines as well as in glioblastoma (T98G) cells [24]. The same two compounds discussed earlier were also the most cytotoxic ones in these cell lines, with the HeLa cells showing the highest sensitivity [24]. 

The anti-cancer and antioxidant properties of other phytochemical ingredients from *S. rebaudiana* were recently evaluated by in vitro and in silico studies [60]. Specifically, six bioactive secondary metabolites were isolated and identified using spectroscopic methods. Dried and powdered fresh leaves were extracted with methanol, then filtered and evaporated. An aliquot of methanol extract was fractionated by n-hexane, dichlormethane or ethyl acetate and the resultant fractions were also evaporated. Subsequently, the ethyl acetate and n-hexane fractions were further dissolved in methanol, mixed with small amount of silica gel and subjected to silica column chromatography. The anti-cancer effects of the four compounds isolated from the ethyl acetate fraction (5-*O*-caffeoyl quinic acid, syringin, luteolin and apigenin) and of the two compounds isolated from the n-hexane fraction (jhanol and jhanidiol) were evaluated in HeLa cells. All compounds except of luteolin showed an anti-proliferative effect in HeLa cells in a dose-dependent manner, as evaluated using the MTT cell viability assay. The anti-cancer drug methotrexate (IC_50_ 80.1 μΜ) was used as a positive control. The most effective compounds were 5-*O*-caffeoyl quinic acid and syringin with IC_50_ values of 0.51 mM (181.3 μg/mL) and 0.52 mM (194.4 μg/mL), respectively. A molecular docking in silico study was also conducted with widely used software packages (PyRx, PyMoL 2.3, Discovery Studio 4.5, and Swiss PDB viewer). Molecular docking studies are very important for drug discovery, as they can predict the molecular interactions between a protein and a ligand that are bound together [61]. Through this bioinformatic analysis, a number of possible conformations/orientations can be generated and the free energy of each conformation can be calculated in order to predict the optimized conformation that shows the optimal binding affinity [61]. The compounds in this study were bound to dihydrofolate reductase (DHFR), a protein that was chosen based on previous evidence showing that its inhibition is important in the development of anti-cancer drugs [62] or to glutathione reductase, a major antioxidant enzyme, which is a target for some anti-cancer agents [63]. All the isolated compounds exhibited higher binding affinity to the DHFR compared to the standard drugs ciprofloxacin and methotrexate. Similarly, all the isolated compounds exhibited a better affinity to the glutathione reductase compared to the reference agent ascorbic acid. The 5-*O*-caffeoyl quinic acid showed the highest DHFR and glutathione reductase binding affinity among the compounds tested [60]. This in silico study supported the idea that the high structural stability of these compounds might explain their potent inhibitory effect.

The anti-proliferative and antioxidant effects of the dried ethanolic extract from *S. rebaudiana* produced by Lopez et al. (also see Section 3.2) were also investigated in HeLa cells, along with stevioside [46]. Both the extract and stevioside showed a dose-dependent activity, but the former exhibited a higher cytotoxic effect. Stevioside displayed cytotoxicity when used at 71 μg/mL (88 μΜ), whereas only 48 μg/mL of the ethanolic extract was sufficient to decrease survival of HeLa cells by 50% [46]. It is noteworthy that the HeLa cells were more sensitive to the extract than the GI cancer cell lines discussed in the previous section. 

Plant-derived essential oils are composed primarily of terpenes and exhibit a wide range of bioactivities, including anti-cancer properties [64,65]. It has been reported that the essential oil of stevia leaves is a complex mixture of mono- and sesqui-terpenes, and their oxygenated derivatives [66]. The in vitro cytotoxicity of the fractioned essential oil from the flowering twigs of *S. rebaudiana* was evaluated for the first time by Mann T S et al. [67]. In this study, 43 oil components, making up 95.5% of it, were characterized using chromatographic and spectroscopic techniques. Gas chromatography revealed that the 10 major compounds of the oil were (*E*)-caryophyllene (15.9%), bicyclogermacrene (14.6%), β-pinene (12.5%), α-humulene (6.6%), germacrene D (6.1%), linalool (5.5%), (Z)-β-farnesene (4.2%), epi-α-cadinol (3.9%) caryophyllene oxide (3.9%) and trans-nerolidol (3.8%), composing 77.7% of the oil. These compounds were tested and showed promising cytotoxic effects in two rat cancer cells lines (C-6; rat glioma cells and CHOK1; Chinese hamster ovary cells) [67].

All previous work described above refers to *S. rebaudiana* derivatives and the research data are also summarized in Table 4. We also found a recent study on the effects of *Stevia pilosa* and *Stevia eupatoria* on cancer cell proliferation and migration with interesting results that are worth reporting. In this study, the effects of a *Stevia pilosa* and a *Stevia eupatoria* methanolic root extract (SPME and SEME, respectively) were evaluated alone, as well as in combination with enzalutamide, on the cell viability and migration of prostate cancer cells [68]. Enzalutamide, an inhibitor of the androgen receptor pathway, has been approved as a hormonal therapy for castration-resistant prostate cancer [69]. The researchers used the wound-healing assay in order to evaluate the migratory capacity of androgen-dependent and androgen-independent prostate cell lines (LNCaP and PC-3, respectively) after 24, 48 and 72 h. Both extracts were not cytotoxic in human fibroblasts in concentrations under 1000 μg/mL, whereas they significantly reduced the viability of prostate cancer cells in all concentrations used (250–3000 μg/mL), suggesting that they may be a potential source of molecules for the treatment of cancer. Treatment with stevia extracts in combination with enzalutamide resulted in greater inhibition of the proliferation and migration of prostate cancer cells compared to the extracts alone, but not compared to enzalutamide alone [68].

## 4. Discussion

In recent years, we have witnessed a substantial rise in cancer incidence, especially in tumor types associated with lifestyle risk factors, such as a diet rich in processed food and poor in plant-derived nutrients [2]. Part of a disease-promoting diet is also the increased intake of sugar, which has been associated with obesity, an independent risk factor for breast and colorectal cancer, but has also been implicated in inflammation and oxidative stress, which are linked to a multitude of malignancies [70]. Therefore, adopting a dietary pattern with a reduced sugar intake could serve as a primary means for cancer prevention [71]. 

The plant *Stevia rebaudiana Bertoni* can be a valuable component of such a diet, since it is a non-caloric sweetener. This property of stevia is based on the way SGs are metabolized within the human body, a process which generates glucose molecules that do not enter the bloodstream [3]. Consequently, Stevia can play an important role in cancer prevention by minimizing the glucose blood levels, thus, alleviating the above-mentioned risk factors—obesity, inflammation, oxidative stress—but also by cutting down on an essential nutrient supply for tumor cells. 

Additionally, numerous studies highlight the anti-cancer activity of bioactive stevia compounds, stemming from the regulation of crucial functions in tumor cells. In this review we have surveyed the related literature and reported the most important findings regarding the biological activities of stevia derivatives in in vivo and in vitro cancer models.

In general, stevia products, including the core aglycone steviol, steviol glycosides, chemically synthesized or modified derivatives of the above, as well as various extracts, manifest potent cancer cell growth inhibitory effects. It is of particular interest that several groups report cytotoxic effects comparable to those exhibited by conventional anti-cancer drugs, such as 5-FU, and one study has even reported less toxicity on non-cancerous cells, making stevia products attractive candidates with prophylactic and restorative function in the disease. However, only a couple of studies have addressed the issue of safety and efficacy of these promising compounds in in vivo models; this crucial parameter should be thoroughly examined by future research projects, before we are able to confidently label stevia products as “cancer preventive and therapeutic” agents.

The mechanisms mediating stevia cytotoxicity include mainly cell cycle arrest, through the regulation of key proliferation proteins, and induction of the apoptotic process. These phenomena have been described by most groups and in different cell lines; therefore, they can be considered the primary mode of action of stevia metabolites in cancer cells. Inhibition of DNA replication enzymes and regulation of important miRNAs by stevia metabolites have also been described. However, an in-depth mechanistic analysis that will identify the primary targets of at least the mono-constituent substances in stevia, i.e., the SGs, and the way they function in the cells is still missing.

At the same time, it has become evident, based on the experimental data reviewed herein, that stevia derivatives and especially its extracts can exhibit their anti-cancer effects not only directly, by being cytotoxic compounds themselves, but also indirectly, through their antioxidant and lipid regulation capabilities. The polyphenols and flavonoids contained in the leaf extracts can protect the cell components from oxidative damage and thus reduce the risk of oxidative stress leading to tumor development. Importantly, the antioxidative capacity of various stevia products was shown by several groups using different methodologies, it can, therefore, be convincingly regarded as an essential antitumor property of the plant. The role of stevia derivatives in targeting the mevalonate pathway has been described only in one recent report; however, the results are compelling and should be further pursued.

Another noticeable trait in the studies described herein is that most of them have used stevioside and its hydrolysis product isosteviol, perhaps understandably, since the former is the most abundant SG. However, there are more than 40 SGs known and one cannot exclude the possibility that other SGs may also possess even more potent anti-cancer activities; future research efforts could be aimed towards their isolation and evaluation in appropriate models. 

In conclusion, *Stevia rebaudiana Bertoni* has great potential not only as a sweetener, but also as a food additive that promotes a wide variety of health benefits. It can be an integral part of a plant-based diet that is used for cancer prevention or therapy in combination with conventional treatments. Nevertheless, further research is required to determine the daily intake of stevia products that is both safe and health promoting in the human body.

## Figures and Tables

**Table 1:** Polarity of solvents used (according to [33] and online sources) and IC_50_ values of the stevia extracts described in [32].

	Solvent	Extract IC_50_ (μg/mL)
**Polarity Decreases** 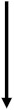	Water	374
	Methanol	228
	Ethanol	180
	Acetone	150
	Chloroform	100
	Petroleum Ether	79

**Table 2:** Biological effects of *S. rebaudiana* derivatives in breast cancer models.

Cell Line/Model	Compound	Effect (Cell Viability Assay)	Reference
F344 rats	Stevioside	Decrease in mammary adenomas	[21]
MCF-7	Steviol	G2/M arrest, ROS-mediated apoptosis (SRB Assay)	[22]
	Stevioside	G1 arrest, Bax overexpression, apoptosis (MTT Assay)	[23]
	Hydroalcoholic extract	Increased cytotoxicity (MTT Assay)	[36]
	Stevia extracts (various solvents)	Increased cytotoxicity (SRB Assay)	[32]
	ZnS Nanoparticles, aqueous extract	Increased cytotoxicity (MTT Assay)	[42]
	Ammonium derivatives of steviol and isosteviol	Increased cytotoxicity (Multifunctional Cytell Cell Imaging system)	[24]
MCF-7, MDA *	Aqueous extract	Decreased cell viability (MTT Assay)	[37]
MDA-MB-231	Steviolbioside	Cell proliferation inhibition (MTT Assay)	[27]
	Isosteviol Triazole Conjugates	Increased cytotoxicity (MTT Assay)	[28]
MDA-MB-231, SKBR3	Stevioside	Cell proliferation inhibition (MTT Assay)	[25]

* no further description.

**Table 4:** Biological effects of *S. rebaudiana* in other types of cancer.

Model/Cancer Type	Compound	Effect (Cell Viability Assay)	Reference
A549 (Lung)	Steviol, isosteviol derivatives	Increased cytotoxicity (MTT Assay)	[44]
	Isosteviol Triazole Conjugates	Increased cytotoxicity (MTT Assay)	[28]
	Ammonium derivatives of steviol and isosteviol	Increased cytotoxicity (Multifunctional Cytell Cell Imaging system)	[24]
BALL1 (Leukemia)	Stevioside and isosteviol derivatives	Increased cytotoxicity (MTT Assay)	[45]
C-6 (Rat glioma)	Flowering twigs essential oils	Increased cytotoxicity (SRB Assay)	[67]
CHOK-1 (Chinese hamster ovary)	Flowering twigs essential oil	Increased cytotoxicity (SRB Assay)	[67]
HeLa (Cervix)	Isosteviol Triazole Conjugates	Increased cytotoxicity (MTT Assay)	[28]
	Stevioside and ethanolic extract	Increased cytotoxicity (MTT Assay)	[46]
	Secondary metabolites from leaves (except luteolin)	Increased cytotoxicity (MTT Assay)	[60]
	Ammonium derivatives of steviol and isosteviol	Increased cytotoxicity (Multifunctional Cytell Cell Imaging system)	[24]
HL-60 (Leukemia)	Steviol and isosteviol derivatives	Increased cytotoxicity and apoptosis (MTT Assay and Flow cytometry Analysis)	[44]
	Isosteviol Triazole conjugates	Effect on cell proliferation (CellTiter-Glo^®^ Luminescent Cell Viability Assay)	[28]
MOLT-4 (Leukemia)	Stevioside and isosteviol derivatives	Increased cytotoxicity (MTT Assay)	[45]
	Isosteviol Triazole conjugates	Effect on cell proliferation (CellTiter-Glo^®^ Luminescent Cell Viability Assay)	[28]
PC-3 (Prostate)	Isosteviol Triazole Conjugates	Increased cytotoxicity (MTT Assay)	[28]
	Ammonium derivatives of steviol and isosteviol	Increased cytotoxicity (Multifunctional Cytell Cell Imaging system)	[24]
T98G (Glioblastoma)	Ammonium derivatives of steviol and isosteviol	Increased cytotoxicity (Multifunctional Cytell Cell Imaging system)	[24]
U2OS (Osteosarcoma)	Steviol	Increased cytotoxicity, G1 arrest apoptosis (MTT Assay and Flow Cytometry Analysis)	[59]
Mouse skin papillomas	Stevioside, Rebaudiosides A and C and Dulcoside A	Tumor inhibition (TPA/DMBA-induced carcinogenesis)	[56]
	Steviol, Stevioside and Isosteviol	Tumor inhibition (TPA/DMBA-induced carcinogenesis)	[57]
